# The Mathematics of a Successful Deconvolution: A Quantitative Assessment of Mixture-Based Combinatorial Libraries Screened Against Two Formylpeptide Receptors

**DOI:** 10.3390/molecules18066408

**Published:** 2013-05-30

**Authors:** Radleigh G. Santos, Jon R. Appel, Marc A. Giulianotti, Bruce S. Edwards, Larry A. Sklar, Richard A. Houghten, Clemencia Pinilla

**Affiliations:** 1 Torrey Pines Institute for Molecular Studies, 11350 SW Village Parkway, Port St. Lucie, FL 34987, USA; E-Mails: rsantos@tpims.org (R.G.S.); mgiulianotti@tpims.org (M.A.G.); mgiulianotti@tpims.org (R.A.H.); 2 Torrey Pines Institute for Molecular Studies, 3550 General Atomics Court, San Diego, CA 92121, USA; E-Mail: jonappel@tpims.org; 3 Department of Pathology and Center for Molecular Discovery, University of New Mexico, 700 Camino de Salud, Albuquerque, NM 87131, USA; E-Mails: BEdwards@salud.unm.edu (B.S.E.); LSklar@salud.unm.edu (L.A.S.)

**Keywords:** combinatorial libraries, mixture-based libraries, harmonic mean mixture model, mathematical modeling, formylpeptide receptors

## Abstract

In the past 20 years, synthetic combinatorial methods have fundamentally advanced the ability to synthesize and screen large numbers of compounds for drug discovery and basic research. Mixture-based libraries and positional scanning deconvolution combine two approaches for the rapid identification of specific scaffolds and active ligands. Here we present a quantitative assessment of the screening of 32 positional scanning libraries in the identification of highly specific and selective ligands for two formylpeptide receptors. We also compare and contrast two mixture-based library approaches using a mathematical model to facilitate the selection of active scaffolds and libraries to be pursued for further evaluation. The flexibility demonstrated in the differently formatted mixture-based libraries allows for their screening in a wide range of assays.

## 1. Introduction

Mixture-based combinatorial libraries, reviewed in [1,2,3], are an efficient and effective way to explore large, dense areas of the chemical space in an exponentially smaller number of samples In a positional scanning mixture-based combinatorial library, mixtures are systematically arranged and tested in order to determine those most likely to contain active compounds [4,5]. These data are then used to deconvolute the library by making the individual compounds from the functionalities of the most active mixtures. Recent advances in the numerical modeling of mixture-based combinatorial libraries [6] has led to a greater understanding of how the Harmonic Mean model, in conjunction with the presence of multiple structural analogs within each mixture, leads to the differentiation of mixtures containing active compounds from those that do not. Such models have also led to impressive estimates of the robustness of a mixture’s activity to variations in the equimolarity of that mixture’s constituent compounds [7]. Over the last 20 years the number of new positional scanning libraries, including scaffolds comprised of peptides, peptidomimetics, heterocycles, and other classes of small molecules, has increased and the total number of samples available for testing is in the thousands. 

**Figure 1:**
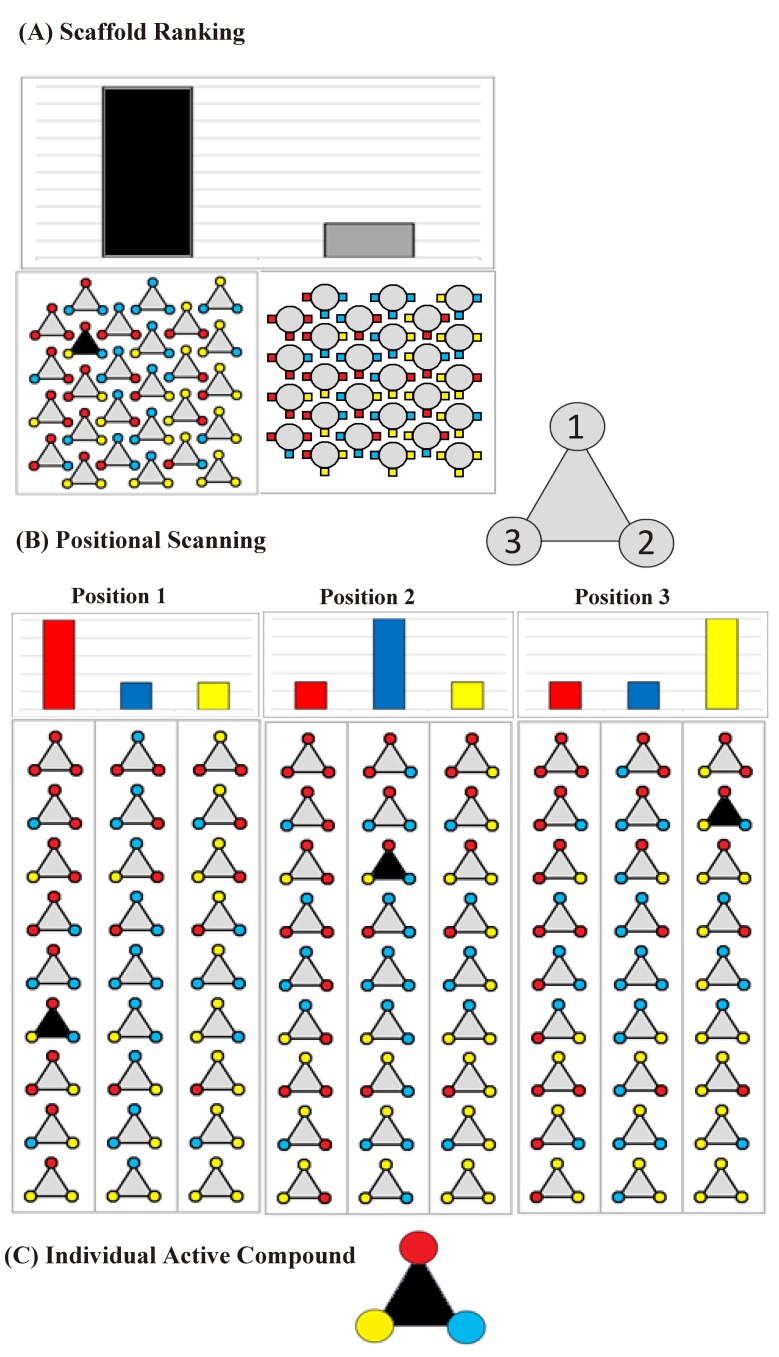
A schematic tracing an active compound (black) through the combinatorial library screening process, from scaffold ranking (**A**) to positional scanning (**B**) to individual compound synthesis (**C**).

In an effort to further increase efficiency and utility as this collection of libraries increases, we previously developed a strategy termed scaffold ranking for the rapid identification and ranking of active library scaffolds [3]. Figure 1 shows a simplified illustration of the screening process using mixture-based combinatorial libraries. In a scaffold ranking library, all compounds in the library are simultaneously present as a mixture in a single sample; Figure 1(A) shows two 27-compound scaffold ranking library samples, with the colors red, blue and yellow representing three choices of functionality at each of three positions. In general, scaffold ranking library samples can result from mixing the cleaved products of the complete positional scanning library or may be synthesized directly as a single mixture. The objective of using scaffold ranking libraries is to prioritize library scaffolds for future analysis, including positional scanning; as shown in Figure 1(A,B), the scaffold which includes the black active compound (represented by a triangle) is chosen for positional scanning because its scaffold ranking mixture is relatively more active when compared to the other scaffold shown (represented by a circle). Only a single positional scanning library is then tested [Figure 1(B)] and deconvoluted (by picking the most active mixtures at each position) in order to find the active compound [Figure 1(C)]. This process can be advantageous in low-throughput assays that would make numerous positional scanning library screenings impractical. The relative efficacy of the scaffold ranking approach provides clear support for its use in low-throughput assays, or costly assays including in vivo screening. The format in which scaffold ranking or positional scanning libraries are used in a particular lead discovery effort will depend on the resources and throughput of the assay. The flexibility of these two screening formats of mixture-based libraries represents a clear advantage for the rapid identification of active lead compounds.

The use of both positional scanning libraries and scaffold ranking have been previously reported [3,8,9,10,11], but there has heretofore never been a comprehensive study comparing scaffold ranking results to positional scanning results across a large number of libraries. In particular, because screening all positional scanning libraries may not be practical in all assays, determining the information both present and absent in a scaffold ranking, relative to positional scanning, is vital for proper usage of the scaffold ranking approach.

**Figure 2:**
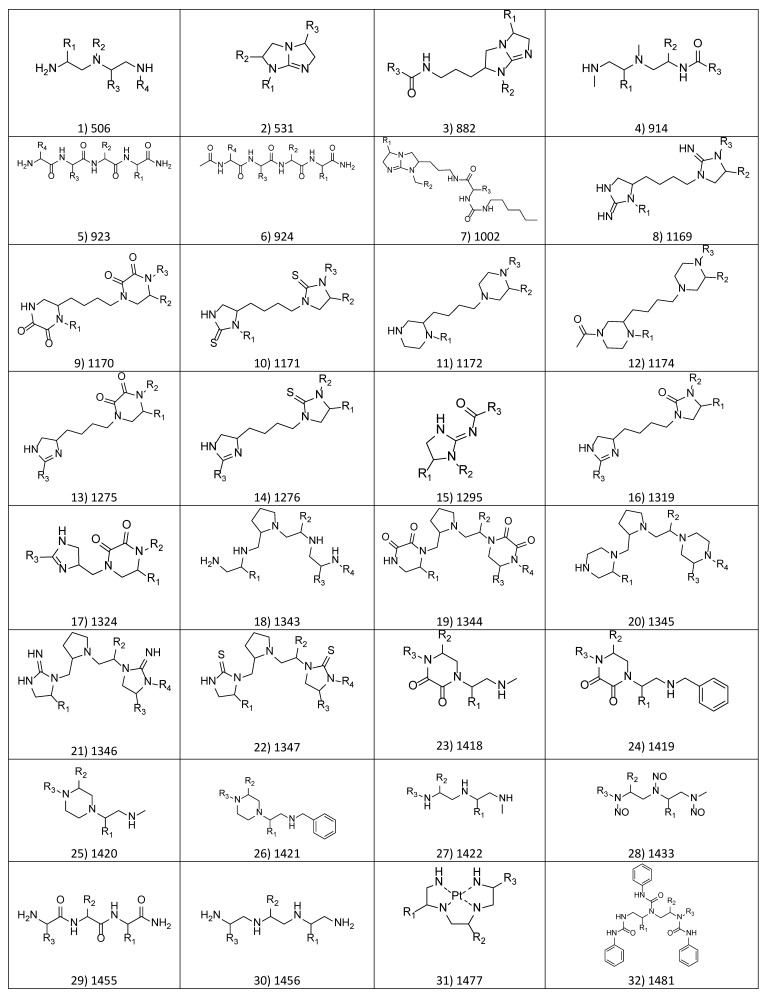
Thirty two small-molecule libraries tested against FPR1 and FPR2.

Herein, we present such a study based on the screening of mixture-based libraries against the formylpeptide receptor (FPR1) and formylpeptide-like1 receptor (FPR2) targets, two receptors that have been implicated in both cancer [12] and inflammatory responses [13]. Thirty-two positional scanning libraries (Figure 2 and Table S1) were tested in their entirety (for a total of 4,304 samples), along with the corresponding 32 scaffold ranking samples. Detailed methodologies and analyses of structures and activities of the individual compounds discovered in this campaign will be presented elsewhere [14,15]. In this study, we present and demonstrate quantitative tools that analyze and use the information present in a positional scanning library screening most important to increasing the likelihood of a successful deconvolution. We also focus on comparing and contrasting the scaffold ranking and positional scanning screening approaches from a mathematical modeling perspective. The results presented here demonstrate that the scaffold ranking library samples lead to effective selection of active positional scanning libraries; consequently, determining the relative activities of the libraries as the first step of a screening campaign does not require the use of the complete collection of positional scanning libraries. This strategy greatly reduces the time and resources required by testing a fraction of the samples with equivalent accuracy. However, it will be also shown that use of the complete collection of positional scanning libraries provides screening data that offers important information, beyond activity alone, which increases the likelihood of the successful deconvolution of a library.

## 2. Results and Discussion

### 2.1. Comparison of Scaffold Ranking and Positional Scanning Using the Harmonic Mean

A positional scanning library is systematically arranged so that, at each position of diversity, every individual compound in the library appears in exactly one mixture in an approximately equimolar fashion. Because of this, an equimolar combination of all of the mixtures in one position of a positional scanning library will result in an equimolar mixture of all the compounds within that library, *i.e.*, the scaffold ranking mixture associated with that library. As previously described, the Harmonic Mean Model accurately describes the activity of a mixture given the activity of its constituents in a simple independent binding assay, such as the data in this study [6]. Thus, for a positional scanning library the harmonic mean model would suggest that:


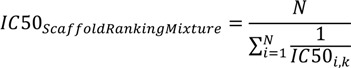
(1)

Here *IC*50_*i,k*_ is the *IC*50 of the *i^th^* mixture of the *k^th^* position, with *N* total functionalities, of a positional scanning library, and *IC*50_*ScaffoldRankingMixture*_ is the *IC*50 of the scaffold ranking mixture associated with that library.

In a high throughput screening (HTS) assay, it is not typical to have dose-response curves for all samples. Such was the case in this study, in which each positional scanning library sample was tested for inhibition of a fluorescent ligand binding to either FPR1 or FPR2 in duplicate at 10 μg/mL and averaged [14]. Each of the scaffold ranking samples was tested in duplicate at 10 μg/mL and 5 μg/mL. Because the Harmonic Mean Model uses the IC_50s_ of samples, it was necessary to extrapolate IC_50s_ for samples using the equation:

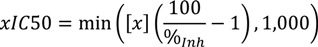
(2)
Here [*x*] is the concentration tested and %*_Inh_* is the percent inhibition value obtained at that concentration. Because %*_Inh_* values close to zero (or negative) could result in arbitrarily high (or negative) *xIC*50 values, 1,000 (*i.e.*, the value obtained when %*_Inh_* ≈ 1% at [*x*] = 10 was chosen as an upper bound. Obviously, these values are extrapolations and not a substitute for actual experimentally determined *IC*50*s*, but they are sufficient for order-of-magnitude estimation.

**Figure 3:**
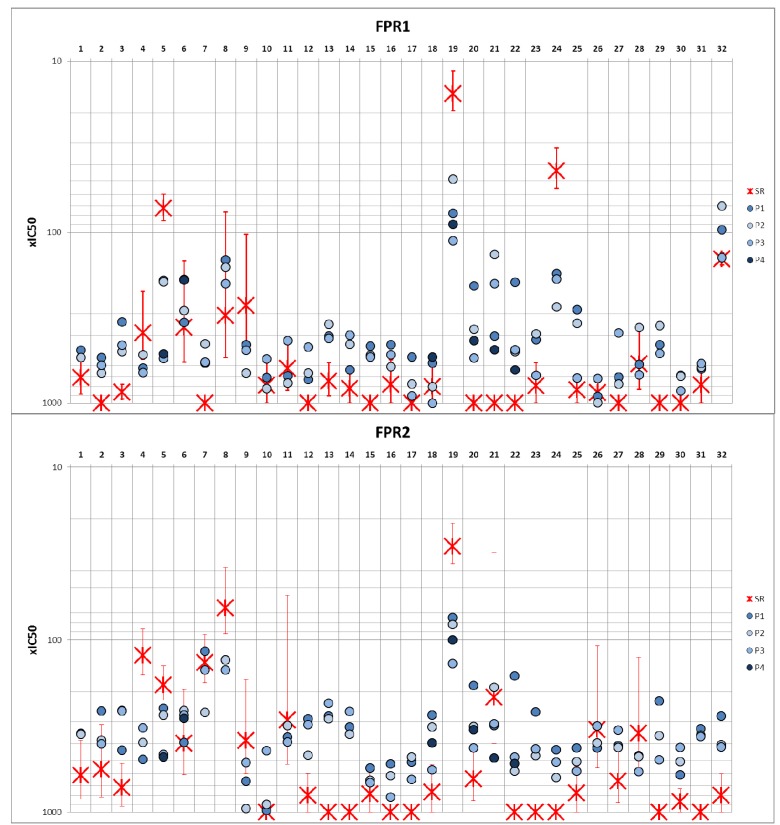
Comparison of the extrapolated scaffold ranking *IC*50 of each library (SR, shown as red stars), and the harmonic means of the extrapolated *IC*50*s* of each position of the positional scanning libraries samples (P1, P2, P3 and P4, shown as blue circles).

All averaged percent inhibition data from all positions of all the positional scanning libraries tested were converted to *xIC*50*s* and the harmonic mean was taken by position for both receptor targets. The four measured percent inhibitions for each scaffold ranking sample were converted to *xIC*50*s* and their average and standard error were calculated. The results are illustrated in Table 1 and Figure 3. The most immediate observation is that the most active library, 19, is detected equally well using either technique; when purely viewed as a method of determining the most potentially active scaffold, using the scaffold ranking libraries is equally effective to using the positional scanning libraries but requires testing of less than one percent of the samples (32 samples *versus* 4,304 samples). 

**Table 1:** Scaffold Ranking *xIC*50*s*, compared to the Harmonic Means of Positional Scanning *xIC*50*s*. Library 19 (red) is the most active in both.

Library	FPR1							FPR2						
	Scaffold Ranking		Harmonic Means of Positional Scanning xIC50s					Scaffold Ranking		Harmonic Means of Positional Scanning xIC50s				
	xIC_50_	SEM	P1	P2	P3	P4	AVG	xIC_50_	SEM	P1	P2	P3	P4	AVG
**1**	708	175	489	540	NA	NA	514	609	226	349	353	NA	NA	351
**2**	1000	0	540	666	601	NA	602	563	252	259	381	402	NA	347
**3**	862	85	334	500	458	NA	431	720	200	437	257	260	NA	318
**4**	389	166	623	521	662	NA	602	123	37	494	393	324	NA	404
**5**	73	13	191	195	547	515	362	183	41	250	273	463	478	366
**6**	361	214	336	287	188	190	250	399	206	393	257	273	284	302
**7**	1000	0	583	451	575	NA	536	136	42	117	264	149	NA	177
**8**	308	232	145	160	200	NA	168	65	27	131	131	149	NA	137
**9**	268	165	456	666	491	NA	538	384	215	660	952	516	NA	709
**10**	786	214	707	820	551	NA	692	1000	0	977	901	440	NA	773
**11**	628	216	690	764	431	NA	628	291	236	365	315	392	NA	358
**12**	1000	0	725	666	470	NA	620	798	202	287	467	311	NA	355
**13**	744	165	406	344	417	NA	389	1000	0	276	288	234	NA	266
**14**	823	177	638	452	400	NA	497	1000	0	321	354	260	NA	312
**15**	1000	0	462	523	538	NA	508	781	219	555	652	672	NA	626
**16**	779	221	456	612	520	NA	529	1000	0	525	615	819	NA	653
**17**	1000	0	538	773	909	NA	740	1000	0	510	478	646	NA	545
**18**	803	197	581	799	1000	538	730	764	236	274	319	566	396	389
**19**	15	4	77	49	112	90	82	29	8	74	82	137	100	98
**20**	1000	0	206	368	545	431	387	640	218	184	319	424	331	315
**21**	1000	0	405	134	200	488	307	215	184	314	188	306	485	323
**22**	1000	0	197	500	487	640	456	1000	0	162	577	479	521	435
**23**	790	210	424	394	685	NA	501	1000	0	262	469	430	NA	387
**24**	44	12	174	273	188	NA	212	1000	0	433	627	512	NA	524
**25**	839	161	284	341	711	NA	445	771	229	424	508	579	NA	504
**26**	871	129	912	994	717	NA	874	332	223	424	397	316	NA	379
**27**	1000	0	705	772	389	NA	622	660	218	411	420	334	NA	388
**28**	588	242	593	361	684	NA	546	349	223	469	475	581	NA	508
**29**	1000	0	455	351	510	NA	439	1000	0	227	359	497	NA	361
**30**	1000	0	688	696	849	NA	744	866	134	606	509	421	NA	512
**31**	782	218	630	614	584	NA	609	1000	0	329	357	364	NA	350
**32**	144	12	97	70	141	NA	102	800	200	277	407	418	NA	367

Considering the inherent inaccuracy of single-dose *IC*50 extrapolations one would not expect perfect correspondences between scaffold ranking *xIC*50*s* and the harmonic mean of a position’s *xIC*50*s*. In general, however, scaffold ranking activities corresponded well to those obtained by harmonic meaning each position; only three comparisons resulted in even a four-fold disparity against the average harmonic mean of its positional scanning library, and 41 of the 64 total comparisons had under a two-fold disparity. Many differences were the result of the scaffold ranking *xIC*50 being 1,000, and the positional scanning harmonic means being lower; this is unsurprising, since by imposing a cap on *xIC*50 values, errors would necessarily be one-sided. The three largest deviations, however, were all overestimates: Libraries 5, 19, and 24 against FPR1. Library 19 had the highest error (over five-fold more active than the average harmonic mean of its positional scanning library) but was the most active library against FPR1 in either case. Libraries 5 and 24, while showing above-average activity in their positional scanning samples, were not actually the second- and third- most active libraries against FPR1; library 32, whose scaffold ranking *xIC*50 and positional scanning harmonic means corresponded quite well, was actually the second-most active overall. It should be noted, however, libraries 5 and 24 do not exhibit substantially less active positional scanning harmonic means than library 32.

### 2.2. Analysis of Positional Scanning Profiles

As shown above, scaffold ranking is equally capable of gauging the overall activity of a given library. However, when the assay throughput rate allows, there is a wealth of additional information present in a full screening of all positional scanning libraries that can aid in choosing the most promising libraries to deconvolute. One of the most important aspects of a positional scanning activity profile is the level of activity differentiation of samples at each position. Given the same overall library activity, a positional scanning activity profile that shows few mixtures at each position that are much more active than the rest is likelier to have compounds that are more active than one with little differentiation. To see why this is the case, consider a library with a scaffold ranking sample *IC*50*s* of 100 μM containing inactive compounds with *IC*50*s* of 1,000 μM and an unknown percentage of active compounds of fixed unknown activity. Under the assumptions of the Harmonic Mean model, such a library could theoretically have a composition of compounds ranging from 100% of compounds with *IC*50*s* of 100 μM each, to 0.01% of compounds with an *IC*50 of 11 nM each, to even smaller percentages of even more active compounds. If, in such a library, a position contained only one mixture that exhibited activity higher than that of an inactive compound (therefore being a well-differentiated profile), then that mixture would need to have a very high relative activity (so that the harmonic mean of that position would come out to 100 μM), and thus the vast majority of the active compounds would be mathematically required to be within that mixture. Since that mixture represents only a fraction of the total library, this in turn puts an upper bound on the percentage of active compounds that could be in the library; as presented above, the lower the percentage of active compounds, the greater the required activity of each active compound. In contrast, if a position contained mixtures all with approximately the same activity, then these mixtures’ *IC*50*s* must be approximately 100 μM each in order for their harmonic mean to be 100 μM. Thus each mixture would be required to have approximately the same number of active compounds, and so no upper bound can be placed on the overall percentage of active compounds.

In an effort to quantify the activity profile of a positional scanning library position that models activity differentiation, the following procedure was developed. For a given position with *n* functional groups, let 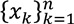 be the rank-ordered activities of the mixtures in that position, so that *x*_1_ is the most active mixture’s activity, *x*_2_ is the second-most active mixture’s activity, etc. In this study, percentage inhibition values were used for the activities; since we are attempting to compare the differentiation of positional scanning profiles within a single study, absolute scaling issues are irrelevant so long as they are consistent, and so long as higher numbers correspond to greater activity. Next, the maximum drop in activity:

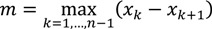
(3)
was calculated. This represents the maximum sequential activity difference within the position; clearly, the more difference between active and inactive mixtures, the greater *m*. The value of *k* for which the largest drop occurs, *K*, is calculated as well: 


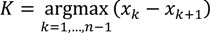
(4)

For an ideally differentiated positional scanning library activity profile, then, one would see high activity differences between active and inactive mixtures (*i.e.*, a high value of *m*) in a relatively small number of mixtures (*i.e.*, a low value of *K*). To this end, the *index of differentiation* of a positional scanning position’s profile is defined as:


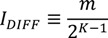
(5)

**Figure 4:**
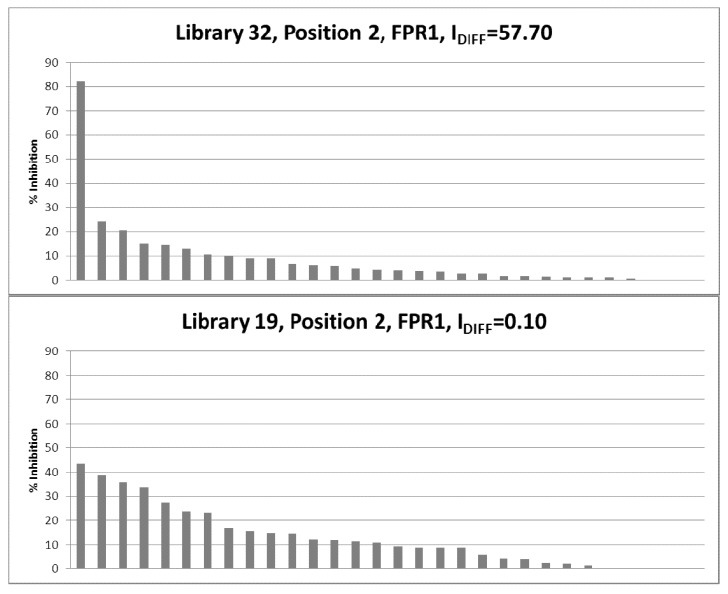
Examples of very high differentiation (Library 32, Position 2, for FPR1) and little differentiation (Library 19, Position 2, for FPR1) in positional scanning profiles, as defined in Equation (5). Note that overall, Library 19 exhibits more activity, but Library 32 is clearly more well-differentiated. Additional zero percent inhibition values have been removed from Library 32’s profile for clarity.

The values of *I_DIFF_* for each position of each of the 32 libraries in this study are shown in Table 2. Selected profiles illustrating high and low differentiation are shown in Figure 4. Note that *I_DIFF_* can vary greatly from position to position in a given library; this is unsurprising, since specific functionalities at certain positions will inevitably be more important to the activity potential of a compound than others. Library 32 exhibited by far the highest average index of differentiation for FPR1, having the highest single position *I_DIFF_*, and two remaining positions ranking 6th and 11th. Libraries 20, 21, and 24 showed relatively high differentiation in some positions, but not all, and had the next highest average *I_DIFF_*. For FPR2, library 19 had the highest average *I_DIFF_*, followed closely by libraries 20 and 29; all three exhibited high-ranking differentiation in two of their positions.

**Table 2:** Indices of Differentiation and Deconvolutability for the 32 libraries against both targets. The most differentiated positions and the most deconvolutable libraries are shown in red.

Library	FPR1						FPR2					
	I_DIFF_					I_DECON_	I_DIFF_					I_DECON_
	P1	P2	P3	P4	AVG		P1	P2	P3	P4	AVG	
**1**	4.55	1.01	NA	NA	2.78	5.41	2.55	2.05	NA	NA	2.30	6.56
**2**	3.70	0.65	0.80	NA	1.72	2.85	10.60	1.00	1.40	NA	4.33	12.47
**3**	0.15	1.00	1.30	NA	0.82	1.90	1.80	4.10	0.55	NA	2.15	6.77
**4**	3.60	3.30	1.70	NA	2.87	4.76	4.50	1.40	0.09	NA	2.00	4.95
**5**	6.00	0.26	0.15	6.10	3.13	8.64	0.80	0.38	0.70	0.13	0.50	1.37
**6**	1.85	15.20	14.10	4.70	8.96	35.82	1.65	4.60	15.70	9.50	7.86	26.03
**7**	0.09	1.15	1.40	NA	0.88	1.64	9.10	0.26	10.95	NA	6.77	38.33
**8**	0.07	0.58	0.00	NA	0.21	1.27	8.30	0.68	0.44	NA	3.14	22.89
**9**	1.80	1.50	1.00	NA	1.43	2.67	1.05	0.15	2.20	NA	1.13	1.60
**10**	0.90	2.30	7.00	NA	3.40	4.91	0.60	3.70	10.50	NA	4.93	6.38
**11**	0.04	2.70	0.00	NA	0.91	1.45	0.03	0.43	0.70	NA	0.38	1.07
**12**	1.30	0.00	0.00	NA	0.43	0.70	0.17	0.12	3.80	NA	1.36	3.84
**13**	0.18	2.85	0.58	NA	1.20	3.09	1.45	0.00	5.05	NA	2.17	8.14
**14**	0.48	0.00	3.55	NA	1.34	2.70	4.85	4.80	0.23	NA	3.29	10.57
**15**	0.00	0.11	2.05	NA	0.72	1.42	0.03	0.53	0.31	NA	0.29	0.46
**16**	1.65	0.16	0.83	NA	0.88	1.66	2.60	0.95	0.20	NA	1.25	1.91
**17**	2.75	1.85	5.23	NA	3.28	4.43	3.60	1.80	0.00	NA	1.80	3.31
**18**	0.05	2.55	0.00	0.44	0.76	1.04	2.05	0.00	0.29	0.00	0.58	1.50
**19**	15.23	0.10	5.38	1.29	5.50	67.09	41.45	36.85	0.74	0.18	19.81	201.44
**20**	25.05	0.85	0.90	19.70	11.63	30.00	36.35	0.00	0.01	23.50	14.96	47.54
**21**	4.25	43.85	0.00	0.63	12.18	39.72	0.03	24.95	1.15	0.14	6.57	20.30
**22**	8.38	0.02	1.70	1.75	2.96	6.49	13.78	0.56	2.75	1.05	4.53	10.43
**23**	3.65	0.01	1.30	NA	1.65	3.30	0.01	0.80	2.70	NA	1.17	3.02
**24**	21.95	4.30	13.20	NA	13.15	62.15	2.45	0.02	1.25	NA	1.24	2.36
**25**	4.60	1.70	0.88	NA	2.39	5.37	2.10	0.00	0.01	NA	0.70	1.40
**26**	2.75	0.65	0.07	NA	1.16	1.32	0.93	0.04	1.50	NA	0.82	2.17
**27**	0.02	0.46	0.53	NA	0.34	0.54	0.40	0.00	0.02	NA	0.14	0.36
**28**	0.19	0.00	0.85	NA	0.35	0.64	1.70	1.60	0.00	NA	1.10	2.17
**29**	1.45	1.60	0.63	NA	1.23	2.79	41.00	1.45	7.35	NA	16.60	46.00
**30**	2.80	0.09	0.85	NA	1.25	1.67	0.04	0.68	4.50	NA	1.74	3.40
**31**	0.20	0.00	0.80	NA	0.33	0.55	2.30	0.01	2.85	NA	1.72	4.92
**32**	12.35	57.70	18.40	NA	29.48	287.89	3.00	0.74	0.60	NA	1.45	3.94

**Figure 5:**
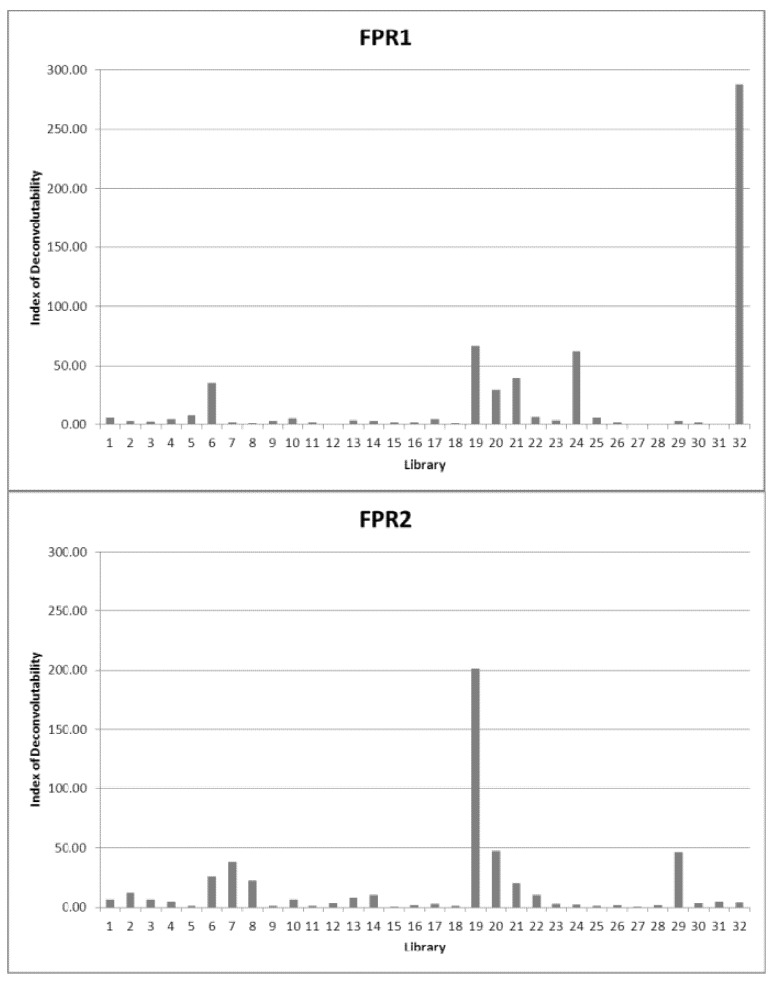
Indices of Deconvolutability for each library, as defined in Equation (6), against both targets.

As reasoned above, high differentiation is very important for potentiating the discovery of highly active compounds in a positional scanning screening profile. Such differentiation in the absence of overall activity, however, may only result in varying degrees of inactive compounds. Therefore, the overall potentiation *index of deconvolutability* of a library is better quantified as:

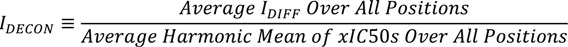
(6)
The values of *I_DECON_* for each library are in Table 2 and graphed in Figure 5. As is evident, each receptor has one standout library: library 32 for FPR1, because of high relative activity and very high relative differentiation, and library 19 for FPR2 (which had the second highest score in FPR1 as well), because of very high relative activity and high relative differentiation. Indeed, these libraries were the two chosen in this study for deconvolution, and both proved to lead to the identification of highly active individual compounds with nanomolar K_i_ values [14].

### 2.3. Selectivity in Scaffold Ranking and Positional Scanning

**Figure 6:**
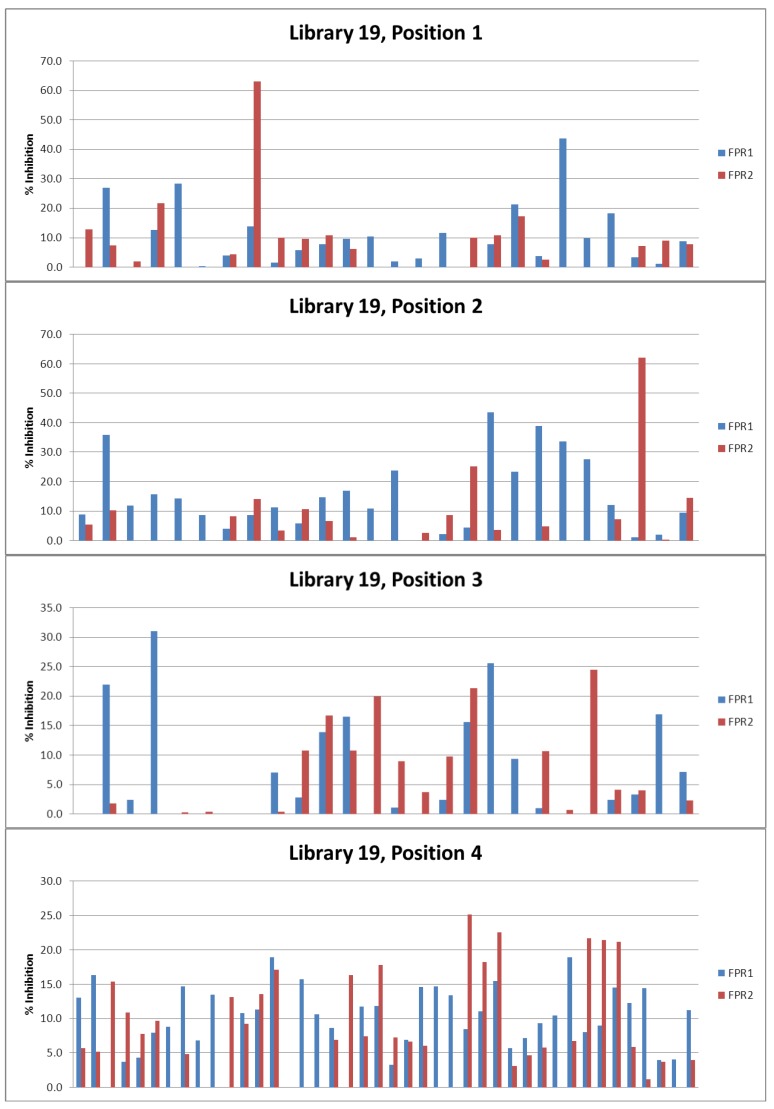
The full positional scanning profile of Library 19. Notice that there are many instances of different mixtures among the most active at the FPR1 target not being active at theFPR2 target, and vice versa. This indicates the potential selectivity that was eventually found.

In the event that selectivity is a desirable endpoint in a study, as it was in this study, additional important lessons can be learned about the relative utility of screening scaffold ranking libraries versus complete positional scanning libraries. As has already been noted, library 19 showed the highest level of overall scaffold ranking activity in both receptors. Library 32, in contrast, only showed substantial scaffold ranking activity against the FPR1 target. Using this information to infer that library 19 could not include selective compounds, however, would not be an appropriate use of the activity of the scaffold ranking samples. The absence of activity in FPR2 for library 32 did indeed imply, both in its positional scanning profile and its eventual deconvolution, an absence of FPR2-active individual compounds. The reverse, however, proved not to be true, as is evident from a closer inspection of library 19’s positional scanning activity profile (Figure 6). Although library 19 exhibits overall high activity against both targets, the mixtures at each position that exhibit that activity vary greatly; FPR2 shows greater differentiation in the first two positions (as evidenced by its higher index of differentiation as described above), and the mixtures of maximum activity do not correspond to those of FPR1. These patterns persisted when individual compounds were tested. Thus, positional scanning libraries should be selected and screened even if the scaffold ranking screening does not show the desired selectivity. Positional scanning libraries offer a window into the possibility of additional selectivity of individual compounds that would not be evident in the analysis of the scaffold ranking library’s activity alone.

## 3. Conclusions

In the past, scaffold ranking has been used as a first step for determining which library will be tested using positional scanning. With the side-by-side data presented in this study, we have shown for the first time that scaffold ranking is indeed sufficient for accurately demonstrating the overall activity of a library, with each library presenting essentially the same activity levels in its scaffold ranking format as in its full positional scanning format. However, we have also demonstrated that, when feasible, complete screening of all positional scanning libraries allows for additional analyses of the differentiation and selectivity that can drastically increase the likelihood of a successful deconvolution. If only the scaffold ranking samples had been tested, library 19 surely would have been chosen, based on the basis of its activity, to screen the complete positional scanning library; as we have shown in this study, to exclude a library on the grounds of selectivity using only scaffold ranking information is a mistake. The potential of identifying selective compounds is only revealed from analysis of its positional scanning profile. As will be presented in a complementary study, 106 individual compounds were synthesized and tested from library 19 [14]. Nineteen compounds had Ki values ≤ 100 nM for FPR1, of which 15 were FPR1 selective (Ki values for FPR2 are more than 100-fold greater); 23 compounds had Ki values ≤ 100 nM for FPR2, of which 12 were selective for FPR2. Furthermore, Library 32, with less activity exhibited in the scaffold ranking than other libraries, may not have been explored at all, had its impressively differentiated profile not been determined through screening its positional scanning library. Deconvolution of library 32 resulted in the synthesis of only eight individual compounds, of which four had Ki values ≤ 20 nM in FPR1 and were highly selective. Additional libraries (library 24 for FPR1, and libraries 20 and 29 for FPR2) that have not yet been deconvoluted show about the same indices of deconvolutability as the successfully deconvoluted library 19 for FPR1; these are clearly a possible direction for future research. By having the scaffold ranking data in tandem with the positional scanning data, one is better able to see the strengths and weaknesses of each approach, and use this knowledge to further increase the effectiveness of already-effective mixture-based combinatorial library screening. 

## Supplementary Materials

Supplementary materials can be accessed at: http://www.mdpi.com/1420-3049/18/6/6408/s1.
