# Supplementary material for: The Mathematics of a Successful Deconvolution: A Quantitative Assessment of Mixture-Based Combinatorial Libraries Screened Against Two Formylpeptide Receptors
Source: Molecules. 2013 May 30;18(6):6408–24. doi: 10.3390/molecules18066408 (PMC4106117; doi:10.3390/molecules18066408)
Supplement: Supplementary file 1 [file molecules-18-06408-s001.pdf]

Supplementary Materials

Table S1. 32 small-molecule libraries tested against FPR1 and FPR2.

Number	Library	Samples	Compounds/ mix	Total	Name	Structure
1	506	364	230	42,320	Alkylated triamine	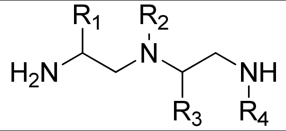
2	531	141	2,009 -2,499	102,459	Bicyclic guanidine	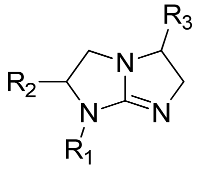
3	882	125	1,681 – 1,763	72,283	C-6-acylamino bicyclic guanidine	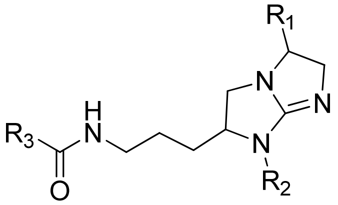
4	914	150	2,500	125,000	N-acyl triamine	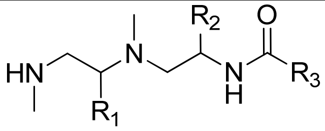
5	923	240	216,000	12,960,000	L-, D-, unnatural Tetrapeptide	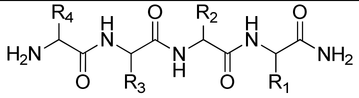
6	924	240	216,000	12,960,000	L-, D-, unnatural Tetrapeptide	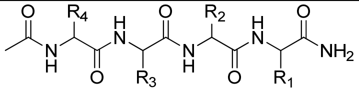
7	1002	109	1,190–1,400	47,600	Urea-linked bicyclic guanidine	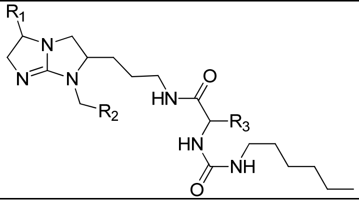
8	1169	110	1,092–1,764	45,864	Bis-cyclic guanidine	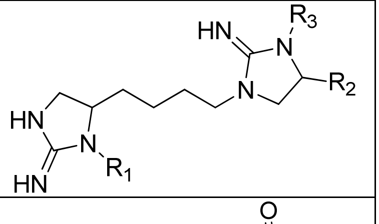
9	1170	110	1,092–1,764	45,864	Bis-diketopiperazine	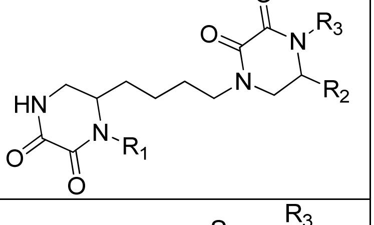
10	1171	110	1,092–1,764	45,864	Bis-cyclic thiourea	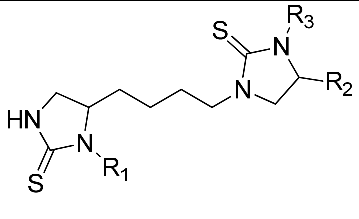

Table S1. *Cont.*

Number	Library	Samples	Compounds/ mix	Total	Name	Structure
11	1172	110	1,092–1,764	45,864	Bis-piperazine	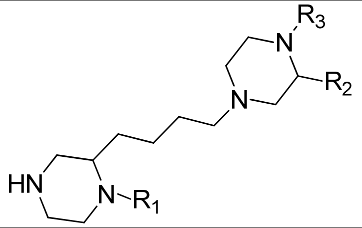
12	1174	110	1,092–1,764	45,864	N-acylated Bis-piperazine	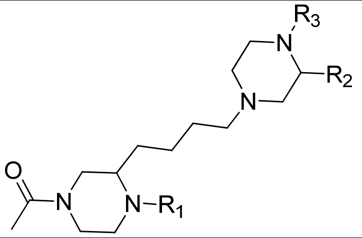
13	1275	116	1,258–1,665	56,610	Dihydroimidazolyl-butyl-diketopiperazine	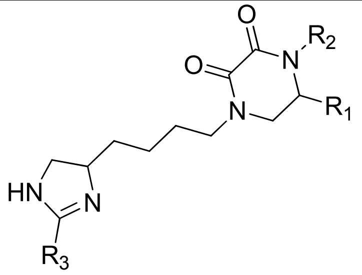
14	1276	116	1,258–1,665	56,610	Dihydroimidazolyl-butyl-cyclic thiourea	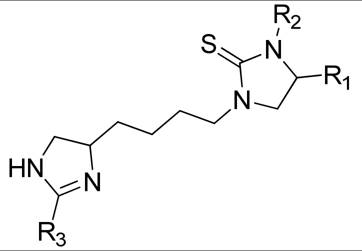
15	1295	107	1,224–1,332	45,288	Acylated cyclic guanidine	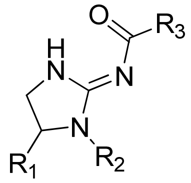
16	1319	116	1,258–1,665	56,610	Dihydroimidazolyl-butyl-cyclic urea	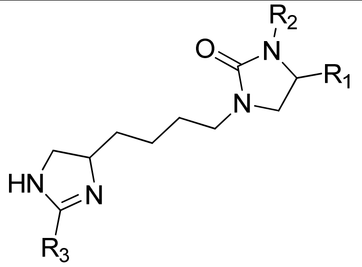
17	1324	116	1,258–1,665	56,610	Dihydroimidazolyl-methyl-diketopiperazine	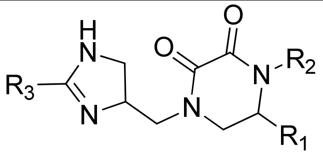

Table S1. *Cont.*

Number	Library	Samples	Compounds/ mix	Total	Name	Structure
18	1343	120	17,576–28,392	738,192	Pyrrolidine pentamine	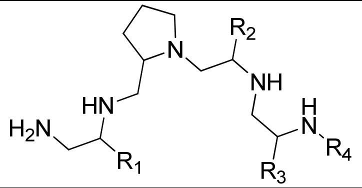
19	1344	120	17,576–28,392	738,192	Pyrrolidine Bis-diketopiperazine	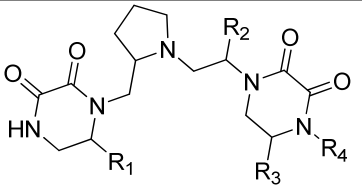
20	1345	120	17,576–28,392	738,192	Pyrrolidine Bis-piperazine	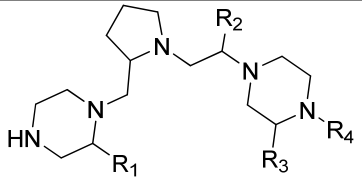
21	1346	120	17,576–28,392	738,192	Pyrrolidine Bis-cyclic guanidine	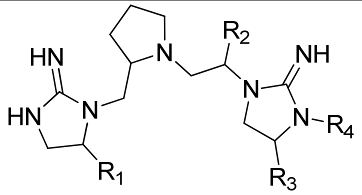
22	1347	120	17,576–28,392	738,192	Pyrrolidine Bis-cyclic thiourea	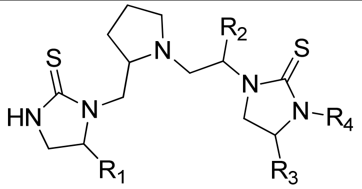
23	1418	96	783–1,160	31,320	N-Methyl-1,4,5-trisubstituted-2,3-diketopiperazine	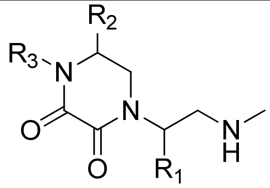
24	1419	96	783–1,160	31,320	N-Benzyl-1,4,5-trisubstited-2,3-diketopiperazine	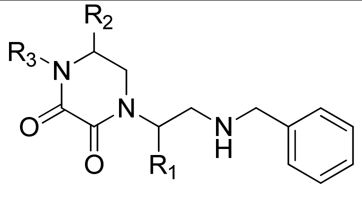
25	1420	96	783–1,160	31,320	N-methylated 1,3,4-trisubstituted piperazine	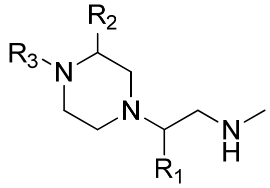

Table S1. *Cont.*

Number	Library	Samples	Compounds/ mix	Total	Name	Structure
26	1421	96	783–1,160	31,320	N-benzylated 1,3,4-trisubstituted piperazine	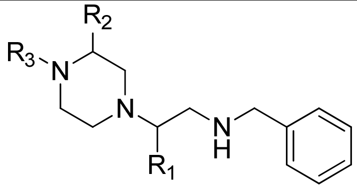
27	1422	96	783–1,160	31,320	N-Methyltriamine	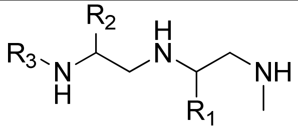
28	1433	74	361–684	12,996	Nitrosamine	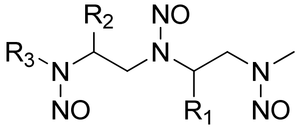
30	1456	174	3,364	195,112	Tetramine	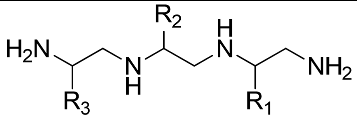
31	1477	174	3,364	195,112	Platinum tetramine	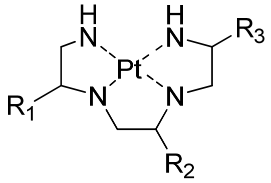
32	1481	135	1,872–2,304	89,856	Poly-phenylurea	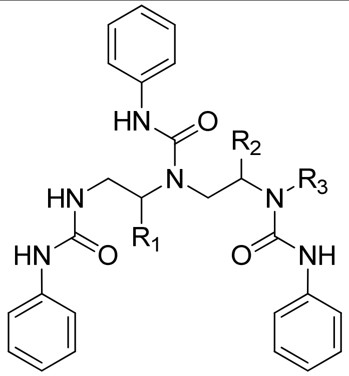
